# Solvent-Free Synthesis, DNA-Topoisomerase II Activity and Molecular Docking Study of New Asymmetrically *N,N'-*Substituted Ureas

**DOI:** 10.3390/molecules171112882

**Published:** 2012-11-01

**Authors:** Andressa Esteves-Souza, Claudio E. Rodrigues-Santos, Catarina de Nigris Del Cistia, Daniel Rosa da Silva, Carlos Maurício R. Sant’Anna, Aurea Echevarria

**Affiliations:** Departamento de Química, Instituto de Ciências Exatas, Universidade Federal Rural do Rio de Janeiro, 23890-000, Seropédica, Rio de Janeiro, Brazil

**Keywords:** ureas, solvent-free synthesis, DNA-topoisomerase, molecular docking

## Abstract

A new series of asymmetrically *N,N'*-substituted ureas **20**–**25** was prepared using solvent free conditions, which is an eco-friendly methodology, starting with Schiff bases derived from cinnamaldehyde and *p*-substituted anilines, which are subsequently submitted to reduction reactions that afford the corresponding asymmetric secondary amines. All of the intermediates were prepared using solvent free reactions, which were compared to traditional methodologies. All of the reactions required a remarkably short amount of time and provided good yields when solvent free conditions were employed compared to other methodologies. The DNA-topoisomerase II-α (topo II-α) activity was evaluated in relaxation assays, which showed that all of the compounds inhibited the enzyme activity at 10 μM, except for urea **24**. Furthermore, a molecular docking study indicated that the compounds **20**–**25** binding to the topo II-α are able to interact with the same binding site as the anticancer drug etoposide, suggesting that the ureas could inhibit the enzyme by the same mechanism of action observed for etoposide, which prevents re-ligation of the DNA strands.

## 1. Introduction

Recently, chemists have become interested in developing methodologies for solvent-free organic reactions to minimise the environmental damage due to the disposal of organic solvents, to reduce reaction times, to increase reactions yields, to reduce costs and to simplify the experimental procedures [1,2]. A solvent-free or solid-state reaction can be performed using the reactants alone or by incorporating them into solid supports. This methodology can also be performed using thermal processes as well as microwave or ultrasound irradiation. Often, the products of solid state reactions may be different from those obtained by reactions in the presence of solvent, due to specific spatial orientation or packing of the reacting molecules in the crystalline state. The interaction of reactants is obtained by simple mixing of the components, allowed to stay at room temperature or heated carefully in an oil bath or exposed to appropriate radiation until the reaction is complete [3,4].

Ureas are well known to be an important class of compounds with a wide range of biological activities, such as antiviral [5,6], antitumor [7,8] and antimicrobial [9,10] activities. Due to the importance of these properties, it is important to synthesise new asymmetrically *N,N'*-substituted ureas, preferably under eco-friendly conditions.

The DNA-topoisomerases are ubiquitous nuclear enzymes that play crucial roles in DNA metabolism events, such as replication and transcription [11]. Because of the crucial role of topoisomerases in the maintenance and replication of DNA during proliferation, cells become highly vulnerable when these functions are lost [12]. 

Our research group has been working on more efficient and cleaner synthetic methods using microwave irradiation and solvent-free conditions [13,14,15,16]. To extend our investigation, we report the synthesis of a new series of asymmetrically *N,N'*-substituted ureas using cinnamaldehyde-derived Schiff bases, which are subsequently reduced and reacted with suitable isocyanates. All the steps were executed under solvent-free conditions with good yields being obtained after short reaction times, which represents a good example of an eco-friendly methodology. Furthermore, the DNA-topoisomerase II inhibitory effect of the final products was evaluated using a relaxation assay, and a molecular docking study was implemented to identify the molecular cause of the observed enzyme inhibition profile.

## 2. Results and Discussion

### 2.1. Synthesis and Spectroscopic Characterisation

**Scheme 1:**
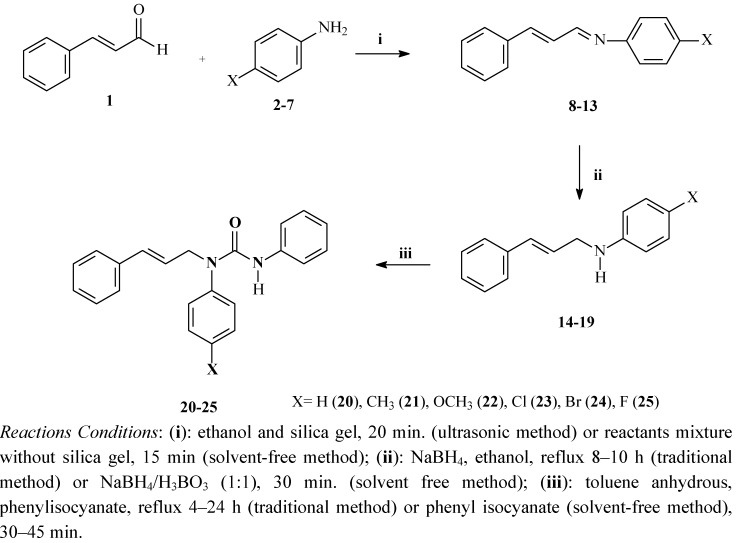
Synthesis of asymmetrically *N,N'*-substituted ureas.

A new series of asymmetrically *N,N'*-substituted ureas **20**–**25** was synthesised using solvent free reactions. The synthetic procedure was performed in three steps starting with Schiff bases derived from cinnamaldehyde with subsequent reduction reactions affording the corresponding allyl amines, which was followed by coupling with phenyl isocyanate, as outlined in Scheme 1. The syntheses of all of the compounds, Schiff bases, corresponding amines and ureas were performed under solvent free conditions.

First, the Schiff bases were prepared from cinnamaldehyde (**1**) and 4-substituted-anilines **2**–**7**, which were mixed using a mortar and pestle for 15 min. The products **8**–**13** were obtained in good yield and were recrystallised from ethanol. The Schiff bases were also prepared with the same reactants, silica gel and ethanol as solvent using an ultrasonic bath for 20 min. The products were obtained after silica gel filtration, solvent evaporation and recrystallisation from ethanol. Table 1 shows the yields and the corresponding melting points of the Schiff bases prepared under both reaction conditions.

**Table 1:** Yields and melting points of the Schiff bases (**8**–**13**) obtained in solvent free conditions and under ultrasonic irradiation.

Schiff base	X	MP (°C)	Ultrasonic irradiation Yield (%) 20 min	Solvent free condition Yield (%) 15 min
**8**	H	107 ^a^	70	100
**9**	4-CH_3_	77 ^b^	70	98
**10**	4-OCH_3_	120–121 ^c^	85	100
**11**	4-Cl	104–106 ^d^	90	97
**12**	4-Br	120–121 ^d^	90	100
**13**	4-F	69–71 ^d^	75	98

^a^ Lit. 107–109 °C [17]; ^b^ lit. 76–77 °C [18]; ^c^ lit. 119–121 °C [17]; ^d^ lit. 105 °C [19]; ^d^ lit. 121 °C [19].

The allyl amines **14**–**19** were obtained by reduction of the Schiff bases using NaBH_4_ as the reducing agent via two different methodologies. Under solvent free conditions, the reduction products were obtained by vigorous mixing of the Schiff bases with NaBH_4_ and H_3_BO_3_ in a mortar and pestle for 30 min. Next, the solids were washed with a NaHCO_3_ solution, extracted with CH_2_Cl_2_ and dried under vacuum affording the corresponding allyl amines in quantitative yields. The second methodology involved equimolar quantities of the Schiff bases and NaBH_4_ with refluxing for 8 to 10 h in ethanol, which was then evaporated under vacuum. Then, the allyl amines were extracted using the method described above to afford the products in good yields (45–75%). Table 2 shows the yields obtained for the allyl amines via both methodologies.

**Table 2:** Allyl-amine **8**–**12** yields obtained under solvent free conditions and with the reflux method.

Allyl amine	X	MP (°C)	Reflux procedure Yield (%) 8–10 h	Solvent free conditions Yield (%) 30 min
**14**	H	Oil ^a^	60	96
**15**	4-CH_3_	Oil ^b^	45	99
**16**	4-OCH_3_	67 ^c^	55	99
**17**	4-Cl	83 ^c^	72	98
**18**	4-Br	86–87 ^c^	75	98
**19**	4-F	60 ^c^	70	99

^a^ Lit. [20]; ^b^ lit. [21]; ^c^ lit. [22].

The asymmetrically *N,N'*-substituted ureas **20**–**25** were synthesised from allyl amines **14**–**19** in the presence of phenyl isocyanate via two different methods. The traditional methodology afforded the target compounds after refluxing for 4 to 24 h in toluene. Under solvent free conditions, the reactants were mixed vigorously for 30–45 min with a mortar and pestle. The solid products were recrystallised from ethanol, and the oil products were purified by column chromatography using CHCl_3_ as the solvent. The ureas were obtained in excellent yield (98–100%) after remarkably low reaction times (30 min) compared to those obtained with traditional methodology (25–64% and 4–24 h). Table 3 shows the yields for the asymmetrically *N,N'*-substituted ureas obtained from both methodologies.

**Table 3:** Asymmetrically *N,N'*-substituted urea (**20**–**25**) yields obtained in solvent free conditions and by the traditional reflux method.

Urea	X	MP (°C)	Reflux procedure Yield (%)	Time (h)	Solvent free conditions Yield (%) 30 min
20	H	oil	26	4	>99
21	4-CH_3_	65–68	30	4	>99
22	4-OCH_3_	oil	40	24	98
23	4-Cl	98–100	64	4	>99
24	4-Br	oil	30	4	>99
25	4-F	75–78	25	4	>99

The formation of the ureas **20***–***25** was observed via infrared spectra. The ν_C=O_ stretching vibration observed at 1654 to 1674 cm^−1^ (Experimental section) indicates the formation of the products because the secondary amines (**14**–**19**) do not absorb in this region. These values are in good agreement with the literature values (1623-1647 cm^−1^) [8].

The ^1^H-NMR spectra of compounds **20**–**25** was employed to identify the three aromatic rings. However, other chemical shifts did not change compared to the amines **14**–**19**. The ^13^C-NMR spectra were of fundamental importance in the characterisation because the chemical shifts of the downfield signals with a δ of 154.29 to 154.43 could be attributed to the carbon atom of the urea carbonyl groups (Experimental section).

### 2.2. DNA-Topoisomerase Assays

**Figure 1:**
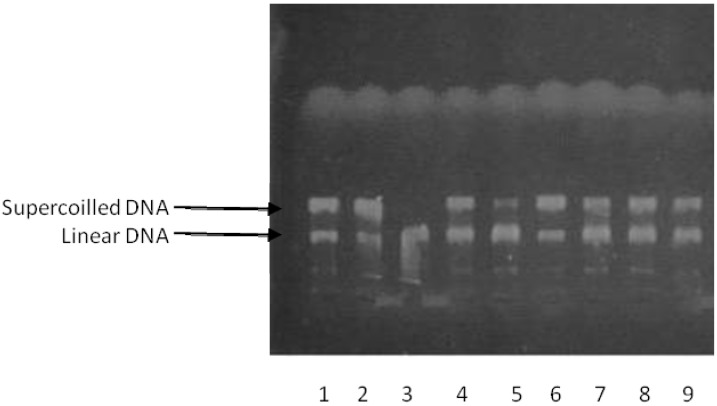
Effect of ureas on topo II-α Line 1: Etoposide (100 μM) + pBR322; line 2: pBR322 only; line 3: pBR322 + topo II-α; line 4: pBR322 + topo II-α + 23 (10 μM); line 5: pBR322 + topo II-α + 24 (10 μM); line 6: pBR322 + topo II-α + 21 (10 μM); line 7: pBR322 + topo II-α + 22 (10 μM); line 8: pBR322 + topo II-α + 20 (10 μM); line 9: pBR322 + topo II-α + 25 (10 μM).

The ability of compounds **20**–**25** to inhibit human DNA topoisomerase II-α (topo II-α) activity was observed in relaxation assays using supercoiled pBR322 plasmid DNA in the presence of ATP. The reaction products were analysed by electrophoretic mobility and revealed in ethidium bromide in the presence of UV light. As shown in Figure 1, all compounds inhibited the enzyme activity at 10 μM; however the urea **24** presented a weak supercoilled DNA band indicating a smaller inhibitory effect. Etoposide, a known topo II-α inhibitor, was used as a positive control at 100 μM.

### 2.3. Molecular Docking Studies

To analyse the molecular details of the mode of interaction with topo II-α, compounds **20**–**25** were docked into the enzyme’s DNA and ATP binding sites. Initially, all of the scoring functions available in the GOLD 5.1 program (CCDC Software Ltd., Cambridge, UK) were tested through a redocking procedure with a co-crystallised ligand (*i.e.*, etoposide and phosphoaminophosphonic acid-adenylate ester, an ATP analogue, the PDB codes for the crystallographic structures are 3QX3 and 1ZXM, respectively). Although all of the available scoring functions were able to dock both ligands into their respective binding sites, the best results were obtained with the ChemPLP function [23,24], which resulted in etoposide and the ATP analogue exhibiting the lowest RMSD values (*i.e.*, 0.250 Å and 0.645 Å, respectively) compared to the crystal structures.

Several poses were obtained for each urea with ChemPLP, and the best-ranked pose for each one was chosen for analysis of the interactions with the enzyme. In the GOLD docking program, the docking functions yield fitness scores, which are dimensionless values. In each case, the scale of the score is a guide of how good the docking pose is with a higher score indicating a better docking result. The fitness scores for each ligand in topo II-α are presented in Table 4.

**Table 4:** Asymmetrically *N,N'*-substituted urea (**20**–**25**), etoposide (ETP) and ATP analogue docking results (ChemPLP scoring function) with the topo II-α DNA and ATP binding sites.

Compound	X	Fitness score into DNA binding site	Fitness score into ATP binding site
20	H	79.54	64.65
21	4-CH_3_	83.94	71.29
22	4-OCH_3_	84.29	69.08
23	4-Cl	82.17	67.01
24	4-Br	80.10	66.90
25	4-F	81.39	69.81
ETP	-	111.26	-
ATP analogous	-	-	143.37

All of the tested ureas were predicted to successfully dock in both binding sites of topo II-α, but the score was higher for the DNA binding site, indicating that this site was the most probable interaction site for these compounds. The differences observed between the scores from the ureas and the ATP analogue also indicated that it would be more difficult for these compounds to effectively compete with ATP by its binding site. In fact, the ATP analogue established a number of interactions that the ureas were not able to establish. Therefore, the docking results suggest that the DNA site in topo II-α is the more probable binding site for this type of molecule, which results in isosteric inhibition. 

**Figure 2:**
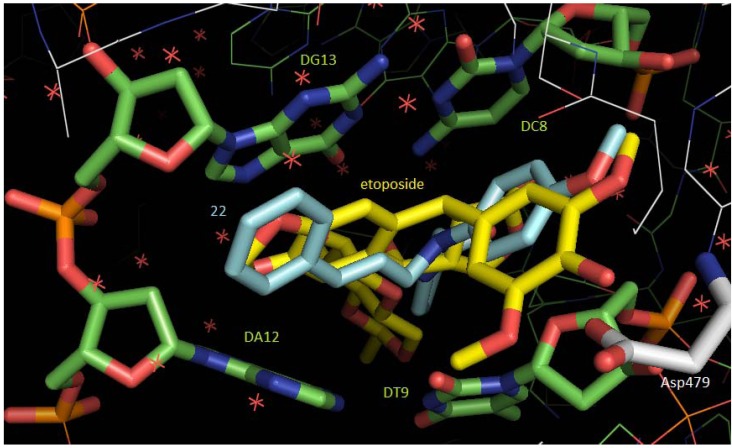
superposition of experimentally observed etoposide (carbon atoms in yellow) interactions and compound **22** (carbon atoms in cyan) docking interactions into the DNA (carbon atoms in green) binding site of topo II-α (carbon atoms in white); H atoms were removed to improve clarity (figure generated with PyMOL software).

Etoposide is known to prevent re-ligation of the DNA strands, and by doing so causes DNA strands to break. Analysis of the co-crystal structure of a fragment of human topoisomerase complexed to DNA and to the anticancer drug etoposide (PDB code 3QX3) revealed that the inhibitor forms H bonds with Asp479 residue and also with a guanine fragment (DG13) of DNA; the inhibitor’s aglicone moiety is inserted between DNA base pairs. Compound **22** was predicted as the best urea ligand for the DNA binding site; although not all interactions observed for etoposide could be matched by compound **22** in the best-ranked docking pose, its overall structure is able to occupy at least partially the same subsites occupied by the co-crystallized ligand molecule. As presented in Figure 2, compound **22** is inserted between the same base pairs as etoposide, which is expected to abolish stacking interactions, effectively blocking religation of the cleaved phosphodiester bond. The major difference is observed in the etoposide’s glycosidic group subsite, but this group is not involved in drug-DNA interactions. The 4-hydroxy-3,5-dimethoxyphenyl and the fused four-ring system of the co-crystallized ligand are mimicked by the methoxy-phenyl and the *N*-[(2*E*)-3-phenylprop-2-en-1-yl]urea groups, respectively, of compound **22**, so a mechanism of action similar to that of etoposide can be proposed for topo II-α inhibition by the urea ligands.

## 3. Experimental

### 3.1. General

The ^1^H- and ^13^C-NMR spectra were obtained on a Bruker Avance II 400 spectrometer (^1^H, 400 MHz; ^ 13^C, 100 MHz) using tetramethylsilane (TMS) as the internal standard and CDCl_3_ as the solvent, and the abbreviations to indicate the multiplicity of ^1^H-NMR spectra signals were: s (singlet), sl (singlet large), d (doublet), dd (double doublet), t (triplet) and m (multiplet). The melting points were determined with a Meltemp II apparatus and were uncorrected. The infrared spectra (KBr pellets) were recorded on a Bruker Vertex 70 spectrophotometer. The ultrasonic-assisted reactions were performed in a Unique ultracleaner bath, model 1400A.

### 3.2. Procedure for the Preparation of N-(p-X-Phenyl)-N-[(E, 2E)-3-phenyl-2-propenylidene]imines ***8**–**13***

#### 3.2.1. Ultrasonic-Assisted Method Using Ethanol as the Solvent

The Schiff bases **8**–**13** were prepared according to previously published methods [25]. The cinnamaldehyde (3.7 mmol), 4-*X*-aniline (3.7 mmol) and silica gel (1 g) were mixed in ethanol (20 mL). The reaction mixture was submitted to an ultrasonic bath for 20 min. Then, the silica gel was filtered, and the products obtained by recrystallised from ethanol and characterised by comparing melting points to the literature values.

#### 3.2.2. Solvent Free Method

The Schiff bases **8–13** were prepared by solid-solid reactions according to previously published methods [26]. The 4-X-aniline (3.7 mmol) and cinnamaldehyde (**1**) were mixed with a mortar and pestle for 15 min. The obtained products were recrystallised from ethanol and characterised by comparing melting points to the literature values.

### 3.3. Procedure for the Preparation of 4-(p-X-Phenyl)-N-[(2E)-3-phenyl-2-propenyl]benzenamines ***14**–**19***

#### 3.3.1. Using Traditional Reflux

NaBH_4_ (2 mmol) was added to a solution of Schiff base (2 mmol) in ethanol (10 mL), and the mixture was refluxed for 8–10 h [8]. Next, water (1 mL) was added, and the products were extracted with CHCl_3_ (3 × 10 mL). The organic phase was dried with anhydrous Na_2_SO_4_, which was followed by filtration. Then, the products were obtained by evaporation under vacuum. The spectra of the obtained products were compared with spectroscopic data obtained from the literature.

#### 3.3.2. Solvent Free Reactions

4-*X*-anilines **2***–***7** and cinnamaldehyde (**1**) (2 mmol each) were vigorously mixed for 15 min in a mortar and pestle. Next, a mixture of NaBH_4_ and H_3_BO_3_ (5 mmol) was added, and the reaction mixture was vigorously mixed for an additional 30 min [26]. Then, the reaction mixture was washed with a NaHCO_3_ solution, and the product was extracted with CHCl_3_ (3 × 10 mL). The chloroform solution was dried with anhydrous Na_2_SO_4_, filtered and evaporated under vacuum. The spectra of the obtained products were compared with spectroscopic data obtained from the literature.

### 3.4. Procedure for the Preparation of Ureas ***20**–**25***

#### 3.4.1. Using Traditional Reflux

A secondary amine **14**–**19** (2 mmol) in anhydrous toluene (30 mL) was mixed with phenyl isocyanate (2 mmol) and refluxed for 4–24 h [8]. The reaction was monitored by TLC (EtOAc–hexane, 7:3). Next, the toluene was evaporated under vacuum. The solid products were recrystallised from ethanol, and the oil products were purified by column chromatography using CHCl_3_ as the eluent.

#### 3.4.2. Solvent Free Reactions

A secondary amine **14**–**19** (2 mmol) and phenylisocyanate (2 mmol) were vigorously mixed for 30–45 min in a mortar and pestle. The time reaction was monitored by TLC (EtOAc–hexane, 7:3). Next, the solid products were recrystallised from ethanol, and the oil products were purified by column chromatography using CHCl_3_ as the eluent.

*N,N'-Diphenyl-N-[(2E)-3-phenylprop-2-en-1-yl]-urea* (**20**). Oil; yield 100%; FT-IR (KBr, υ cm^−1^): 3421 (N-H), 2923/2854 (C-H), 1674 (C=O), 1597/1496 (C=C); ^1^H-NMR (DMSO-*d*_6_): δ 8.02 (s, 1H), 7.41 (d, *J =* 5.0 Hz, 1H), 7.40 (dd, *J =* 5.0 Hz, 2H), 7.38 (d, *J =* 5.0 Hz, 1H), 7.37 (sl, 1H), 7.33 (dd, *J =* 5.0 and 10.0 Hz, 2H), 7.29 (d, *J =* 5.0 Hz, 2H), 7.26 (t, *J =* 5.0 Hz, 2H), 7.20 (t, *J =* 5.0 and 10.0 Hz, 3H), 6.93 (t, *J =* 5.0 and 10.0 Hz, 1H), 6.43 (d, *J* = 15.0 Hz, 1H), 6.34 (dd, *J* = 5.0 Hz and 15.0 Hz) 4.45 (d, *J =* 5.0 Hz, 2H); ^13^C-NMR (DMSO-d_6_): δ 154.43, 142.44, 139.98, 136.46, 131.18, 129.44, 128.65, 128.31, 127.54, 127.28, 126.33, 126.25, 122.14, 119.92, 51.20. Anal. Calcd. for C_22_H_20_N_2_O (328.4071); C, 80.46; H, 6.14; N, 8.53%. Found: C, 81.05; H, 6.02; N, 8.74%.

*N-(4-Methylphenyl)-N-[(2E)-3-phenylprop-2-en-1-yl]urea* (**21**). White solid; m.p. 65–68 °C; yield 99%; FT-IR (KBr, υ cm^−1^): 3419 (N-H), 2920/2854 (C-H), 1658 (C=O), 1595/1519 (C=C); ^1^H-NMR (DMSO-*d*_6_): δ 7.39 (m, *J* = 5.0 and 10.0 Hz, 4H), 7.29 (t, *J* = 5.0 and 10 Hz, 2H), 7.21 (sl, 5H), 7.19 (d, *J* = 5.0 Hz, 2H), 6.92 (t, *J* = 5.0 and 10.0 Hz, 1H), 6.42 ( d, *J* = 15.0 Hz, 1H), 6.33 (dd, *J* = 5.0 and 15.0 Hz), 4.41 (d, *J* = 5.0 Hz, 2H), 2.29 (s, 3H); ^13^C-NMR (DMSO-d_6_): δ 154.43, 140.00, 139.50, 136.48, 135.85, 131.24, 130.06, 128.63, 128.28, 127.45, 126.25, 122.07, 119.88, 51.53, 20.66. Anal. Calcd. for C_23_H_22_N_2_O (342.4337); C, 80.67; H, 6.48; N, 8.18%. Found: C, 80.06; H, 7.04; N, 8.72%.

*N-(4-Methoxyphenyl)-N-[(2E)-3-phenylprop-2-en-1-yl]urea* (**22**). Oil; yield 98%; FT-IR (KBr, υ cm^−1^): 3421 (N-H), 2956/2929/2839 (C-H), 1672 (C=O), 1597 (C=C); 1247/1031 (O-C-O); 1178 (C-CO-O); ^1^H-NMR (DMSO-*d*_6_): δ 7.69 s, 1H), 7.39 (m, *J* = 5.0 and 10.0 Hz, 4H), 7.29 (d, *J* = 5.0 Hz, 2H), 7.25 (d, *J* = 10.0 Hz, 2H), 7.20 (m, *J* = 5.0 and 10.0 Hz, 3H), 6.96 (d, *J* = 10.0 Hz, 2H), 6.92 (t, *J* = 5.0 and 10.0 Hz, 1H), 6.41 (d, *J* = 20.0 Hz, 1H), 6.30 (dd, *J* = 5.0 and 20.0 Hz), 4.37 (d, *J* = 5.0 Hz, 2H) 3.75 (s, 3H); ^13^C-NMR (DMSO-d_6_): δ 157.94, 154.53, 140.02, 136.51, 134.40, 131.37, 129.38, 128.84, 128.63, 128.24, 127.52, 126.26, 122.05, 119.93, 118.22, 114.78, 55.25, 51.68. Anal. Calcd. for C_23_H_22_N_2_O (358.4331); C, 77.07; H, 6.19; N, 7.82%. Found: C, 76.83; H, 6.01; N, 8.04%.

*N-(4-Chlorophenyl)-N-[(2E)-3-phenylprop-2-en-1-yl]urea* (**23**). White solid; m.p. 98–100 °C; yield 100%; FT-IR (KBr, υ cm^−1^): 3431 (N-H), 2922/2852 (C-H), 1654 (C=O), 1595/1527 (C=C); ^1^H-NMR (DMSO-d_6_): δ 8.21 (s, 1H), 7.43 (m, *J* = 5.0 and 10.0 Hz, 4H), 7.38 (d, *J* = 10.0 Hz, 2H), 7.34 (d, *J* = 10.0 Hz, 2H), 7.29 (t, *J* = 5.0 and 10.0 Hz, 2H), 7.21 (t, *J* = 10.0 Hz, 3H), 6.44 (d, *J* = 15.0 Hz, 1H), (dd, *J* = 5.0 and 15.0 Hz, 1H), 4.44 (d, *J* = 5.0 Hz, 2H); ^13^C-NMR (DMSO-d_6_): δ 154.32, 141.33, 139.95, 136.40, 131.38, 130.47, 129.34, 129.10, 128.65, 128.29, 127.60, 126.30, 126.09, 122.27, 120.13, 51.39. Anal. Calcd. for C_22_H_19_ClN_2_O (362.8518); C, 72.82; H, 5.28; N, 7.72%. Found: C, 73.08; H, 4.98; N, 7.93%.

*N-(4-Bromophenyl)-N-[(2E)-3-phenylprop-2-en-1-yl]urea* (**24**). Oil; yield 99%; FT-IR (KBr, υ cm^−1^): 3423 (N-H), 2925/2854 (C-H), 1668 (C=O), 1597/1492 (C=C); ^1^H-NMR (DMSO-d_6_): δ 8.24 (s, 1H), 7.56 (dd, *J* = 5.0 and 10.0 Hz, 2H), 7.40 (m, *J* = 5.0 and 10.0 Hz, 4H), 7.28 (d, *J* = 5.0 Hz, 2H), 7.27 (d, *J* = 5.0 Hz, 2H), 7.21 (m, *J* = 5.0 and 10.0 Hz, 4H), 6.94 (t, *J* = 5.0 and 10 Hz, 1H), 6.44 (d, *J* = 15.0 Hz, 1H), 6.34 (dd, *J* = 5.0 and 15.0 Hz), 4.44 (d, *J* = 6.0 Hz, 2H); ^13^C-NMR (DMSO-d_6_): δ 154.29, 141.81, 139.94, 136.40, 132.26, 131.36, 129.37, 128.65, 128.30, 127.60, 126.52, 126.29, 126.08, 122.28, 120.13, 118.82, 118.23. Anal. Calcd. for C_22_H_19_BrN_2_O (407.3031); C, 64.87; H, 4.70; N, 6.88%. Found: C, 65.02; H, 4.59; N, 7.06%.

*N-(4-Fluorphenyl)-N-[(2E)-3-phenylprop-2-en-1-yl]urea* (**25**). White solid; m.p. 75–78 °C; yield 99%; FT-IR (KBr, υ cm^−1^): 3323 (N-H), 2920/2854 (C-H), 1660 (C=O), 1595/1506 (C=C); ^1^H-NMR (DMSO-*d*_6_): δ 7.99 (s, 1H), 7.40 (d, *J* = 10.0 Hz, 2H), 7.38 (m, *J* = 5.0 and 10 Hz, 4H), 7.29 (t, *J* = 10.0 Hz, 2H), 7.23 (d, *J* = 10.0 Hz, 2H), 7.21 (m, *J* = 5.0 and 10.0 Hz, 3H), 6.93 (t, *J* = 5.0 and 10.0 Hz, 1H), 6.41 (d, *J* = 15.0 Hz, 1H), 6.33 (dd, *J* = 5.0 Hz and 15.0 Hz), 4.42 (d, *J* = 5.0 Hz); ^13^C-NMR (DMSO-d_6_): δ 161.48, 159.55, 154.43, 139.99, 138.38, 136.45, 131.47, 129.96, 129.89, 128.63, 128.25, 127.57, 126.29, 126.13, 122.19, 120.14, 116.29, 116.11, 51.62. Anal. Calcd. for C_22_H_19_FN_2_O (346.3975); C, 76.28; H, 5.53; N, 8.09%. Found: C, 76.84; H, 5.16; N, 8.23%.

### 3.5. Materials for DNA-Relaxation Assays

The DNA-topoisomerase II-α (topo II-α) drug screening kits were provided by TopoGEN [27] and contained supercoiled (form I) plasmid substrate DNA (25 μg in 10 mL TE buffer). TE buffer (10 mM Tris–HCl at pH 7.5 and 1 mM EDTA) and the assay buffer (50 mM Tris–HCl, pH 8, 120 mM KCl, 10 mM MgCl_2_, 100 mM EDTA, 3 mg/mL bovine serum albumin, 0.5 mM dithiothreitol, and 0.5 mM ATP) were used. The supercoiled pBR322 plasmid DNA was purchased from Gibco (Grand Island, NY, USA). The loading buffer contained 25% bromophenol blue, 50% glycerol and 10% SDS. The agarose and the substances utilised in these assays were purchased from Sigma® (St. Louis, MO, USA).

### 3.6. Procedure for the DNA-Topoisomerase Assay

The topo II-α inhibition assay was performed as described in the TopoGEN screening kit. Briefly, the reaction mixture (10 μL) contained the drug, DNA, assay buffer, 2U of topo II-α, and water. In the topo II-α assay, two units were utilised to relax 0.125 μg/mL pBR322 (Gibco). The mixture was incubated at 37 °C for 30 min, and the reaction was terminated by addition of 1 μL of a dye solution containing 25% bromophenol blue, 50% glycerol and 10% SDS (sodium dodecyl sulfate). The products were submitted to electrophoresis using 1% agarose gel in TAE buffer (50× stock: 242 g Tris base, 57.1 mL glacial acetic acid, and 100 mL of 0.5 M EDTA) at 15 V for 3.5 h. The gels were stained with ethidium bromide (0.5 μg/mL) for 30–45 min, washed, and photographed under UV light.

### 3.7. Docking Studies

Compounds **20**–**25** were constructed and energy-minimised with the PM3 semi-empirical method [28] as implemented in the Spartan’08 program (Wavefunction, Inc., Irvine, CA, USA). The resulting structures were used for the docking study of the DNA and the ATP binding sites. Due to the significant structural modifications of the enzyme after ATP binding (and also of its N-substituted analogue) to its specific allosteric site, it was necessary to use different crystal structures for the study of these two binding sites. Docking solutions with the DNA binding site were obtained with the human topo II-α co-crystallised with DNA and etoposide (PDB code 3QX3, resolution 2.16 Å) [29]. For the ATP binding site, human topo II-α co-crystallised with phosphoaminophosphonic acid-adenylate ester, an ATP analogue, in the ATPase domain (PDB code 1ZXM, resolution 1.87 Å) was used [30]. For the docking studies of compounds **20**–**25**, the etoposide and water molecules were deleted from the 3QX3 structure, and the ATP analogue and water molecules were deleted from the 1ZXM crystal structure. Both TOPO-II structures contain Mg^2+^ ions, and the 3QX3 structure contains a co-crystallised DNA segment, which was not removed.

The docking procedure was accomplished with the GOLD 5.1 program (CCDC Software Ltd.), and the hydrogen atoms were added to the protein structures based on ionisation and tautomeric states defined by the program. The number of genetic operations (crossover, migration, mutation) for each run was set to 100,000 in the searching procedure. All of the scoring functions available in the program, such as GoldScore [31], ChemScore [32], ASP [33] and ChemPLP [34], were evaluated and compared for the docking calculations.

The internal binding site for docking was defined with a 20 Å radius from residue Asp479 in the DNA binding site and with a 10 Å radius from the magnesium atom in the ATP binding site. Both are located at the centre of each site. For the docking evaluation and adequate scoring function selection, the co-crystallised structures (etoposide and the ATP analogue, in the respective binding site) were redocked to each original site. The resulting poses of compounds (**20**–**25**) were compared and analysed to identify structural characteristics of the complexes formed by these molecules and topo-II.

## 4. Conclusions

In summary, a new series of asymmetrically *N,N’*-substituted ureas was prepared using solvent free conditions, which is an eco-friendly methodology, for all of the preparation steps involving Schiff bases and the synthesis of the corresponding secondary amines. The synthesised ureas were obtained after remarkably short times and in good yields compared to other methodologies.

The evaluation of the topo II-α activity in the relaxation assays showed that all of the compounds inhibited the enzyme activity at 10 μM, except for urea **24**. The docking results of compounds **20**–**25** into topo II-α indicated that these compounds are able to interact with the same binding site as the anticancer drug etoposide. These results suggest that the same mechanism of action observed for etoposide (*i.e.*, prevents re-ligation of the DNA strands) may also be responsible for the observed enzyme inhibition by the asymmetrically *N,N'*-substituted ureas.
